# Fluid-structure dynamics of a vibro-impact capsule robot in multiphase intestinal environments

**DOI:** 10.1007/s11071-025-12175-z

**Published:** 2026-02-26

**Authors:** Zepeng Wang, Jiyuan Tian, Yang Liu, Ana Neves, Shyam Prasad

**Affiliations:** 1 Exeter Small-Scale Robotics Laboratory, Engineering Department, University of Exeter, Exeter, EX4 4QF UK; 2 Division of Smart Technologies for Tumor Therapy, German Cancer Research Center (DKFZ) Site Dresden, Dresden, Germany; 3 Engineering Department, University of Exeter, Exeter, EX4 4QF UK; 4 Royal Devon University Healthcare NHS Foundation Trust, Barrack Road, Exeter, EX2 5DW UK

**Keywords:** Fluid-structure interaction, Vibro-impact, Finite element, Two-phase flow, Capsule robot

## Abstract

The gastrointestinal tract contains complex fluids, such as mucus, chyme, and water, that can significantly influence capsule robot locomotion by reducing friction or introducing hydrodynamic drag. This study presents a bidirectional fluid-structure interaction model that captures the dynamics of a vibro-impact capsule self-propelling through a fluid-filled small intestine. The model couples the motion of the magnetically actuated capsule, the viscoelastic deformation of the intestinal wall, and a gas-liquid two-phase flow field. Numerical predictions were systematically validated against experimental measurements under controlled laboratory conditions. The results show that an increased liquid volume fraction generates stronger resistance to capsule motion, more so than fluid viscosity alone, by causing fluid accumulation and vortex formation, thereby elevating hydrodynamic pressure and drag. Moreover, capsule performance is improved with higher excitation frequencies and duty cycles, enhancing both propulsion and motion robustness. This work provides a validated numerical platform for designing and optimising magnetically driven capsule robots, advancing their potential for diagnostic and therapeutic applications in the gastrointestinal tract.

## Introduction

**Fig. 1:**
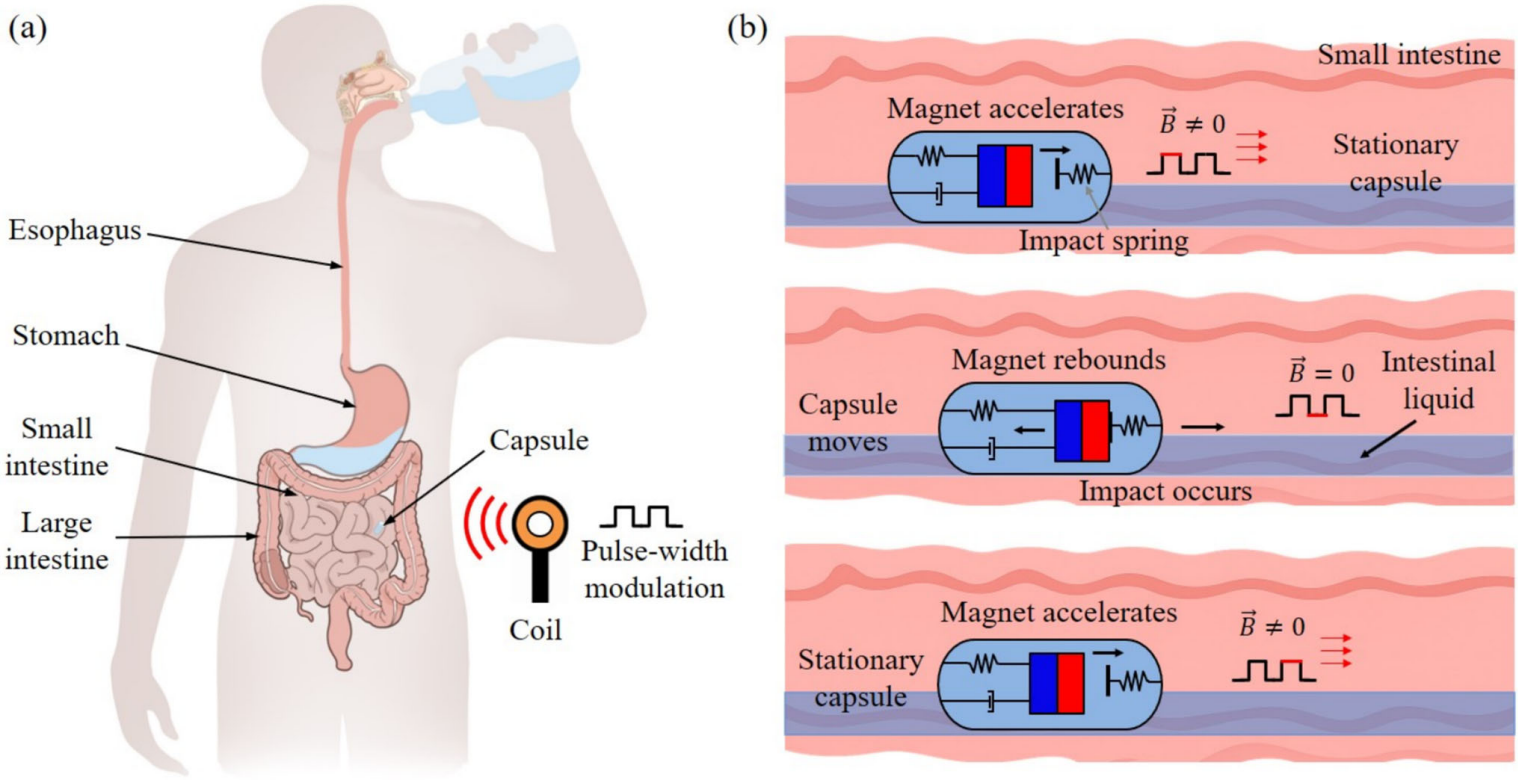
Schematic illustration of the capsule robot system and the working principle of the vibro-impact capsule within the GI tract. (a) The left panel shows the human digestive system, highlighting the pathway of the capsule following oral ingestion of water and bowel preparation fluids. The capsule travels through the esophagus, stomach, small intestine, and large intestine. An external coil placed near the abdomen generates a time-varying magnetic field, controlled via pulse-width modulation, to actuate the capsule wirelessly. (b) The right panel illustrates the operating mechanism of the vibro-impact capsule in the small intestine. When the magnetic field is activated ($\vec {B} \ne 0$), the inner magnet accelerates while the capsule is kept temporarily stationary. Upon deactivation of the magnetic field ($\vec {B} = 0$), the magnet hits the impact spring, producing an impact that propels the capsule forward. After the magnet rebounds, the actuation cycle repeats under alternating magnetic fields, enabling controllable stepwise locomotion in the fluid-filled intestinal environment

In recent years, the global incidence of gastrointestinal (GI) diseases has shown a steady increase, with intestinal cancers now ranking as the second leading cause of cancer-related mortality and the third most prevalent malignant tumour worldwide [1–3]. The conventional diagnostic method, flexible endoscopy, involves inserting a tube-like instrument into the patient’s GI tract [4]. While effective, this approach is often associated with significant drawbacks, including invasiveness, patient discomfort, and a non-negligible risk of trauma or tissue damage [5, 6]. To mitigate these limitations, capsule endoscopy has emerged as a minimally invasive alternative. Equipped with a miniature video camera, these swallowable devices offer a safer and more comfortable means of detecting various GI conditions [7, 8].

To further address the limitations of passive capsule endoscopy, a variety of active capsule robots have been proposed over the past two decades [9], including inchworm-like robots [10, 11], helical robots [12, 13], vibrating capsules [14, 15], and leg-based capsules [16, 17]. However, most of these devices remain at the experimental stage [18], as their protruding appendages pose a potential risk of secondary injury to the GI tract [19]. As illustrated in Fig. 1, our team has previously developed a vibro-impact capsule robot with a smooth exterior, measuring 11 mm in diameter and 26 mm in length [20], designed to enable safer and more effective examination of the small intestine [21]. The capsule consists of a rigid capsule shell, permanent magnet, helical spring and double-sided constraints [22]. Its built-in permanent magnet can be driven by the magnetic field generated by an external handheld coil to oscillate back and forth along the capsule axis and collide with the front and back constraints, thereby driving the capsule forward or backward [23].

Prolonged reciprocation of a capsule at a fixed location within the intestine, as observed in realistic capsule-intestinal contact scenarios, may pose a risk of tissue damage due to sustained mechanical loading [24]. Therefore, understanding the pressure exerted by the capsule during intestinal motility is crucial for ensuring safe operation. Previous studies have primarily focused on capsule-intestine interactions in the absence of surrounding fluid. For instance, Alsunaydih et al. [25] embedded pressure sensors on the surface of a capsule endoscope to measure contact forces during transit. Woo et al. [26] developed a computational model to simulate capsule motion in the small intestine and analysed the spatial distribution of stress components acting on the capsule. Guo et al. [27, 28] examined frictional forces in the intestinal environment, while Tian et al. [29] employed both experimental and finite element (FE) approaches to study capsule-wall interaction forces under varying conditions. These studies underscore the importance of characterising resistance forces, such as contact pressure, friction, and wall stress [30, 31], to inform the design and optimisation of capsule geometry and actuation strategies. A deeper understanding of these mechanical interactions is essential for improving capsule performance, minimising tissue trauma, and enhancing diagnostic reliability.

When applying capsule endoscopy to the human digestive tract, it is essential to consider fluid-related factors that affect capsule mobility [32, 33]. As illustrated in Fig. 1a, patients are typically instructed to ingest large volumes of polyethylene glycol solution and water prior to the examination in order to enhance image clarity and improve diagnostic accuracy [34, 35]. This bowel preparation helps eliminate air bubbles and food residues from the small intestine, thereby facilitating clearer visualisation during capsule transit [36]. However, despite such preparation, prolonged examination times often lead to the reaccumulation of food residues [37] and the presence of highly viscous intestinal fluids [38], which may also arise from pathological conditions [8]. The viscous mucus secreted by intestinal glands and epithelial cells increases hydrodynamic resistance, thereby complicating capsule propulsion within the intestinal lumen [39]. Song et al. [40] reported that intestinal mucus tends to adhere to the intestinal wall, contributing further to flow resistance. Liang et al. [41] conducted experimental studies on liquid-liquid and liquid-solid interactions in intestinal fluid environments, deriving operational performance metrics for capsules under such conditions. Tang et al. [42, 43] investigated flow rate and hydrodynamic resistance around capsules traversing tubes of varying diameters filled with liquid. Similarly, Teng et al. [44] incorporated intestinal wall viscoelasticity and simplified fluid profiles to examine the structural dynamics and stress distributions acting on the capsule.

While previous studies have provided valuable insights into fluid-structure interaction (FSI) relevant to capsule endoscopy, most have relied on simplified motion models or assumed passive capsule dynamics, thereby overlooking the impact of active propulsion and fluid disturbance on capsule behaviour. To address this gap, the present study develops a bidirectional FSI model that captures the actively driven motion of a vibro-impact capsule within a realistic, multiphase intestinal environment. As illustrated in Fig. 1b, the model considers the coupled dynamics between the capsule, the viscoelastic intestinal wall, and the surrounding intestinal fluid. Through a combination of FSI simulations and controlled experiments, we analyse the capsule’s displacement, velocity, fluid interaction forces, and the resulting flow field distributions. These findings provide a foundation for optimising actuation strategies and capsule geometry, with the aim of improving the propulsion efficiency and control of self-propelled capsule robots.

The remainder of this paper is organised as follows. Section 2 presents the governing equations for the fluid and solid domains within the FSI framework, along with a theoretical formulation of the magnetic actuation force generated by the external coil acting on the capsule’s inner magnet. Section 3 introduces the experimental setup developed to evaluate the capsule’s propulsion performance in a fluid-filled artificial intestine, detailing the control scheme and displacement measurement methodology. Section 4 describes the construction and implementation of the FSI model, including material properties, fluid-solid coupling, and three simulation scenarios designed to explore the effects of fluid characteristics, actuation parameters, and capsule geometry. Section 5 compares the experimental results with FSI predictions, validates model fidelity, and provides a detailed analysis of the fluid dynamics associated with capsule motion. Finally, Section 6 summarises the key findings and outlines future directions for improving the propulsion efficiency and control of capsule endoscopes in complex intestinal environments.

## Method

To investigate the fluid dynamics of vibro-impact capsules within the small intestine, this study develops a bidirectional FSI model. The model incorporates the vibro-impact propulsion system, intestinal wall, and surrounding intestinal fluid. Structural dynamics and actuation of the capsule are simulated using the Transient Structural module in ANSYS 2023 R1, while the intestinal fluid, modelled as a gas-liquid two-phase flow, is solved using the Fluent module. These two domains are then coupled to realise a fully bidirectional FSI simulation.

As the capsule traverses the intestinal lumen, the surrounding fluid, composed of both gas and viscous liquid, exerts resistance forces and torques on the capsule. These hydrodynamic effects are captured by resolving the local flow field near the capsule. The intestinal fluid is assumed to be isothermal and incompressible, and its behaviour is governed by the incompressible Navier-Stokes equations [45]. For a Newtonian fluid, the viscous stress tensor is given by $\tau = \mu (\nabla u)$, and the momentum conservation equation becomes

1 $$\begin{aligned} \rho \left( \frac{\partial u}{\partial t} + u \cdot \nabla u \right) = -\nabla p + \mu \nabla ^2 u + f_\text {g}, \end{aligned}$$

where $\rho$ is the fluid density, *u* is the velocity vector, *p* is pressure, $\mu$ is the dynamic viscosity, and $f_\text {g}$ is the body force due to gravity.

In the Arbitrary Lagrangian-Eulerian framework [46], the relative motion between the mesh and the fluid must be considered. The material derivative is used to express the fluid acceleration:

2 $$\begin{aligned} \frac{D u}{D t} = \frac{\partial u}{\partial t} + (u - \vec {v}) \cdot \nabla u, \end{aligned}$$

where $\vec {v}$ is the mesh velocity, and $(u - \vec {v})$ represents the fluid velocity relative to the moving mesh. Substituting this into Eq. (1), the Arbitrary Lagrangian-Eulerian form of the incompressible Navier-Stokes equations becomes

3 $$\begin{aligned}  &   \rho (x, t) \left( \frac{\partial u}{\partial t} + (u - \vec {v}) \cdot \nabla u \right) = -\nabla p\nonumber \\  &   \quad + \nabla \cdot \left( \mu (x, t) (\nabla u + \nabla u^T) \right) + f_\text {g}. \end{aligned}$$

Here, $\rho (x,t)$ and $\mu (x,t)$ denote the spatially varying density and dynamic viscosity of the gas-liquid mixture. These are computed based on the local volume fraction $\alpha$

4 $$\begin{aligned} {\left\{ \begin{array}{ll} \rho (x, t) = \alpha \rho _{\text {liquid}} + (1 - \alpha ) \rho _{\text {gas}}, \\ \mu (x, t) = \alpha \mu _{\text {liquid}} + (1 - \alpha ) \mu _{\text {gas}}. \end{array}\right. } \end{aligned}$$

To capture the evolution of the gas-liquid interface, the convection-diffusion equation is employed for the volume fraction $\alpha$ [47]

5 $$\begin{aligned} \frac{\partial \alpha }{\partial t} + \nabla \cdot (\alpha u) = D \nabla ^2 \alpha , \end{aligned}$$

where *D* is the diffusion coefficient associated with the gas-liquid interface.

The solid domain, representing the capsule structure and intestinal wall, is modelled using the momentum conservation equation in a Lagrangian framework

6 $$\begin{aligned} \rho _\text {s} \frac{\partial ^2 d}{\partial t^2} = \nabla \cdot \sigma + f_\text {g} + F, \end{aligned}$$

where $\rho _\text {s}$ is the density of the solid, *d* is the displacement vector, $\sigma$ is the stress tensor, and *F* represents internal forces generated by the capsule’s propulsion system.

Given that capsule movement occurs at low velocities and under isothermal conditions, thermal effects and heat transfer are neglected. At the fluid-solid interface, coupling is enforced through continuity of velocity and stress

7 $$\begin{aligned} {\left\{ \begin{array}{ll} u = \frac{\partial d}{\partial t}, \\ \sigma _\text {f} \cdot n = \sigma _\text {s} \cdot n, \end{array}\right. } \end{aligned}$$

where $\sigma _\text {f}$ and $\sigma _\text {s}$ are the stress tensors of the fluid and solid, respectively, and *n* is the normal vector to the interface.

## Experimental setup

**Fig. 2:**
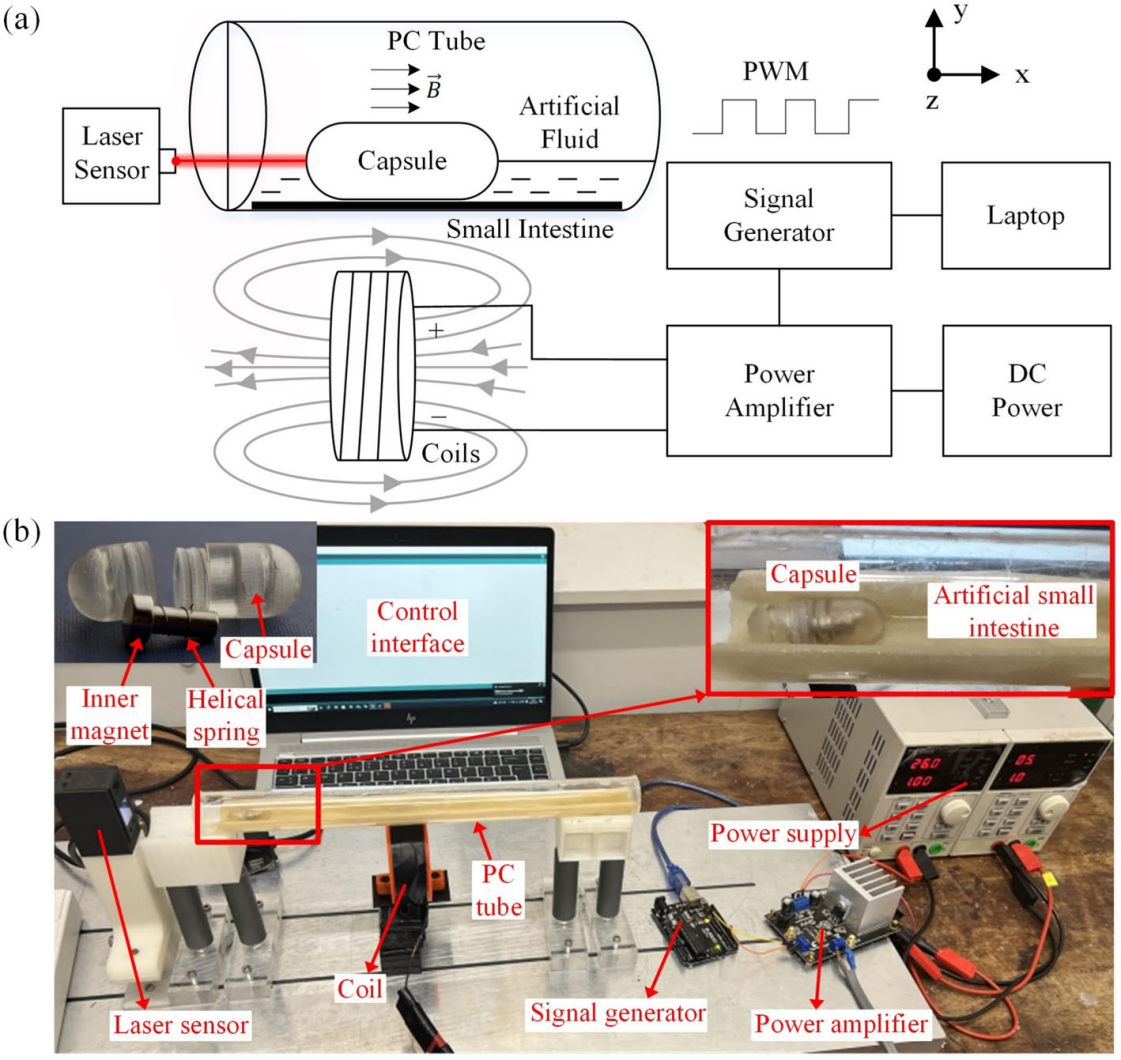
Experimental setup for testing the dynamics of the vibro-impact capsule in the intestinal environment. (a) Schematic diagram of the experimental system, illustrating the arrangement of the capsule inside a fluid-filled PC tube lined with an artificial small intestine. The electromagnetic coil generates a time-varying magnetic field to activate the capsule, while the laser sensor records its displacement. The signal generator produces a PWM control signal, which is amplified and supplied to the coil, all coordinated via a control interface. (b) Photograph of the experimental platform, which includes the key system components: the capsule robot, a laser displacement sensor, an electromagnetic coil, a signal generator, a power amplifier to generate the drive signal, a DC power supply, a PC tube simulating the intestinal tract, and a laptop for control and data acquisition. The inset on the left highlights the capsule prototype, showing the inner T-shaped magnet and the helical spring, while the inset on the right provides a close-up of the capsule robot positioned on the artificial small intestine

To investigate the propulsion characteristics of the vibro-impact capsule within a fluid-filled intestinal environment, a dedicated experimental platform was established, as illustrated in Fig. 2. The system integrates a physical actuation system and a displacement sensing system, enabling precise actuation and robust data acquisition for quantitative analysis.

### Experimental platform

Fig. 2a depicts the schematic diagram of the experimental setup. At the beginning of each experiment, the capsule is placed at a defined starting point in the artificial small intestine within a tube. The external coil is located beneath the capsule, making sure that the axis of the coil is parallel to the capsule axis. The signal generator is used to produce pulse-width modulation (PWM) excitation signals and then the signals are amplified and delivered to the coil to generate a periodic magnetic field on the capsule. The artificial fluid inside the polycarbonate (PC) tube is formulated to replicate the viscosity and flow properties of intestinal fluids, enabling the evaluation of the propulsion performance of the capsule under biomimetic conditions. As the capsule is propelled forward by the magnetic force, a laser sensor is placed at the end of the tube and continuously monitors the capsule’s displacement. The measured data are then acquired by a Data Acquisition Board (NI USB-6210) and transmitted to the laptop. Key experimental parameters, such as excitation frequency, fluid properties, and capsule geometry, are systematically varied to assess their effects on capsule dynamics.

Fig. 2b presents the physical configuration of the experimental setup. The main components include a vibro-impact capsule prototype, an artificial small intestine [48], a PC tube, an external driving coil, a laser displacement sensor, a signal generator, a power amplifier, a DC power supply, and a computer for system control and data recording. Within the setup, the transparent PC tube is partially filled with artificial fluids to emulate the intestinal environment and features an air exchange port at the top. The artificial fluids are made of water and lubricating fluids (Loovara Intimate) in different proportions. The tube length is 305 mm and the inner diameter is 26 mm, which simulates the size of the intestine in expanded mode according to [49]. A layer of artificial small intestine is placed at the bottom of the tube, with the capsule resting on top, facilitating direct observation and measurement. The small intestine is made by combining artificial human tissue analogues and woven fibres, with a length of 300 mm and a thickness of 1 mm. In addition, the capsule shell is 3D-printed from polyethylene material, with a length of 26 mm and a diameter of 11 mm, and the inner magnet is a T-shaped NdFeB magnet. The external driving coil is mounted on a slide rail beneath the PC tube, ensuring better alignment directly underneath the capsule for stable control of the capsule motion. The actuation signal is generated via a signal generator (Arduino Uno), which outputs a PWM signal to the power amplifier. The amplified current is then supplied to the external coil to generate the required magnetic field. A regulated DC power supply provides stable voltage and current for the amplifier and other electronic components.

In order to capture the real-time position and velocity of the capsule, a laser displacement sensor (Omron ZX2-LD100) is positioned at one end of the tube. This experimental platform enables the motion measurement of the vibro-impact capsule under varying magnetic excitation conditions. It also allows for investigation of the effects of the fluid environment on propulsion efficiency and stability and provides essential experimental data for validating the bidirectional FSI numerical simulation models.

**Fig. 3:**
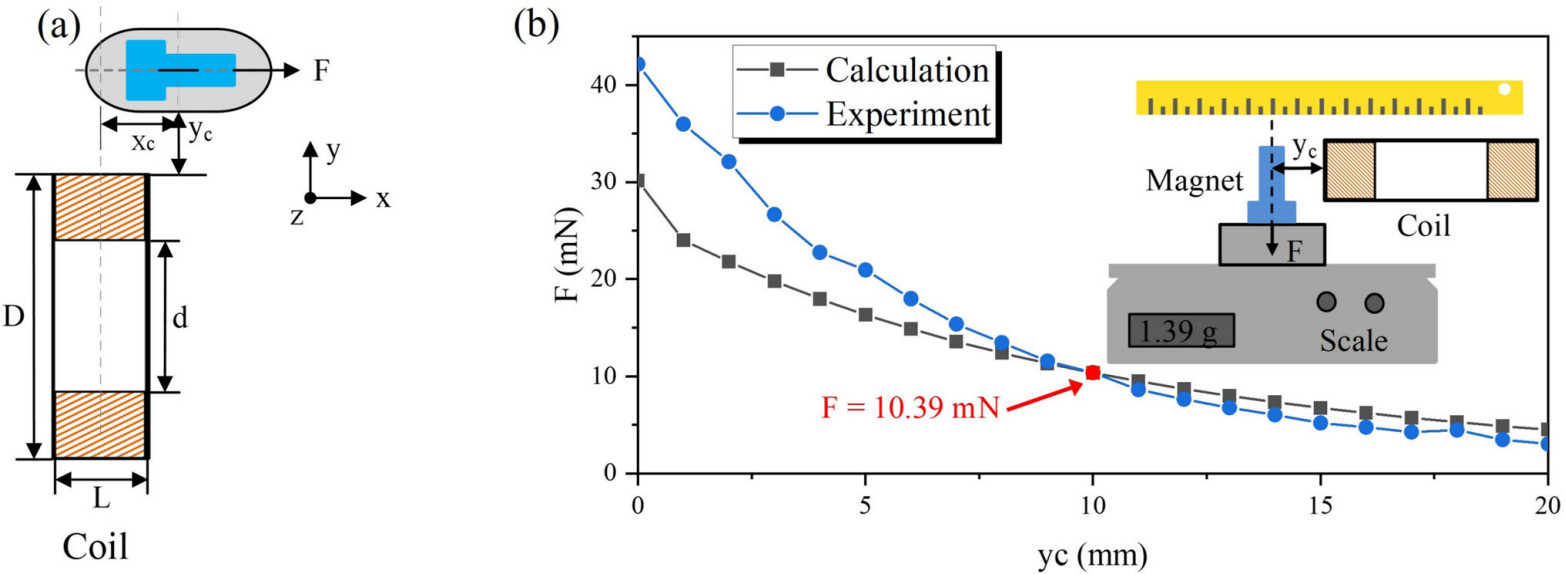
(a) Schematic diagram shows the geometric relationship between the external coil and the capsule. The coil is positioned directly beneath the capsule, with its central axis aligned parallel to the capsule axis. The *x*-axis denotes the primary direction of capsule motion. *D* and *d* represent the outer and inner diameters of the coil, respectively. $y_\text {c}$ is the vertical distance from the coil centre to the capsule’s inner magnet, and $x_\text {c}$ is the horizontal offset between the capsule and the vertical axis of the coil. (b) Experimental apparatus for characterising magnetic driving force and Maxwell simulation results The magnetic force *F* is measured as a function of $y_\text {c}$ using a precision scale. The measured force decreases rapidly with increasing distance from the coil. The red data point highlights the case where $y_\text {c} = 10$ mm, yielding a force of $F = 10.39$ mN, which is used as the baseline condition in subsequent propulsion experiments

### Magnetic force characterisation

To achieve effective propulsion of the capsule within the intestine, the external coil is positioned directly beneath the capsule, with its central axis aligned parallel to that of the capsule, as illustrated in Fig. 2. This setup represents an optimised configuration compared to previous designs using hand-held coils [50]. Since the capsule primarily travels along the horizontal (*x*-) direction, magnetic forces in the vertical (*y*-) and lateral (*z*-) directions are negligible. Under this condition, the horizontal magnetic driving force generated by the coil and acting on the capsule during PWM excitation can be expressed as

8 $$\begin{aligned} F = {\left\{ \begin{array}{ll} v_\text {m} M \dfrac{\partial B_\text {x}}{\partial z}, &  \text {mod}(t, T) \in [0, DT], \\ 0, &  \text {otherwise}, \end{array}\right. } \end{aligned}$$

where $\text {mod}(t, T)$ denotes the modulo operation of time *t* with respect to the signal period *T*, and $D \in (0,1)$ represents the duty cycle. The $v_\text {m}$ is the volume of the inner magnet embedded, and the diameter of the inner magnet head is 7 mm with a length of 3 mm, and the diameter of the tail is 4 mm with a length of 10.2 mm. *M* is its magnetisation intensity at 8.42 $\times$ 10$^5$ A/m, and $B_\text {x}$ is the *x*-component of magnetic induction. In practice, the horizontal displacement of the capsule from the coil axis must be considered, as slight deviations may occur during locomotion. As shown in Fig. 3a, the horizontal offset of the capsule from the coil axis is denoted by $x = x_\text {c}$, and the *x* component of the magnetic field at the location of the capsule can be calculated using the Biot–Savart law [51]

9 $$\begin{aligned} B_\text {x}{=}\frac{\mu _0 I}{4\pi } \int _{0}^{L}\int _{d/2}^{D/2} \int _{0}^{2\pi } \frac{a(y\sin \theta -z\cos \theta )}{[(z-a\cos \theta )^2+(y-a\sin \theta )^2+x_c^2]^{3/2}} \text {d}\theta \text {d}a\text {d}h,\nonumber \\ \end{aligned}$$

where *I* is the current through the coil at 0.716 A, $\mu _0$ is the magnetic constant, the radial integration variable *a* ranges from *d*/2 to *D*/2, *h* is *L*/2, and the angular variable $\theta$ ranges from 0 to $2\pi$. Here $x_\text {c}$ is the axial distance from the centre of the coil to the centre of the inner magnet, *D* and *d* represent the outer and inner diameters of the coil, which are 33 mm and 25 mm respectively, and *L* is the width of the coil at 10 mm. The coil has 910 turns in total.

To experimentally characterise the magnetic driving force, the setup shown in Fig. 3b was employed. The magnetic force acting on the capsule’s internal magnet was measured using a precision scale at varying vertical distances $y_\text {c}$ between the coil and the magnet. The same coil and magnet specifications were used to calculate the theoretical magnetic force using Eq. (8). As shown in Fig. 3b, the calculated and measured forces are in reasonable agreement, particularly around $y_\text {c} = 10$ mm, where the discrepancy is minimal (measured force = 10.39 mN). At shorter distances ($y_\text {c} < 10$ mm), however, the deviation increases, likely due to limitations in the simplified theoretical model or measurement artefacts. Therefore, $y_\text {c} = 10$ mm was chosen as the operating distance in all propulsion experiments to ensure reliable and consistent magnetic force generation.

For the horizontal offset $x_\text {c}$ between the coil and capsule, the coil was manually adjusted during locomotion to remain aligned beneath the capsule, maintaining $x_\text {c}\approx -20$ mm as shown in Fig. 2a. To ensure consistent coil-capsule alignment during each test, the external coil was manually monitored and adjusted by visual observation. Given the slow speed and limited displacement of the capsule, the positional offsets (both axial and vertical) remained minimal throughout locomotion. To reduce variability, each test was repeated five times under consistent alignment conditions.

## Modelling and scenarios

In this study, the three-dimensional bidirectional FSI modelling was established and implemented through ANSYS 2023 R1. This model enables the dynamic coupling between the motion of the vibro-impact capsule and the complex multiphase flow environment within the intestine. The FSI approach incorporates both the transient structure module, describing the vibro-impact capsule moving in the intestine, and the fluid flow module, which employs a Volume of Fluid method to simulate the interactions between the capsule and flows. Furthermore, three representative scenarios were designed to investigate the effects of fluid properties, capsule excitation conditions, and capsule geometry on overall propulsion performance. The following subsections provide a detailed description of each scenario, material properties, and FSI modelling.

**Fig. 4:**
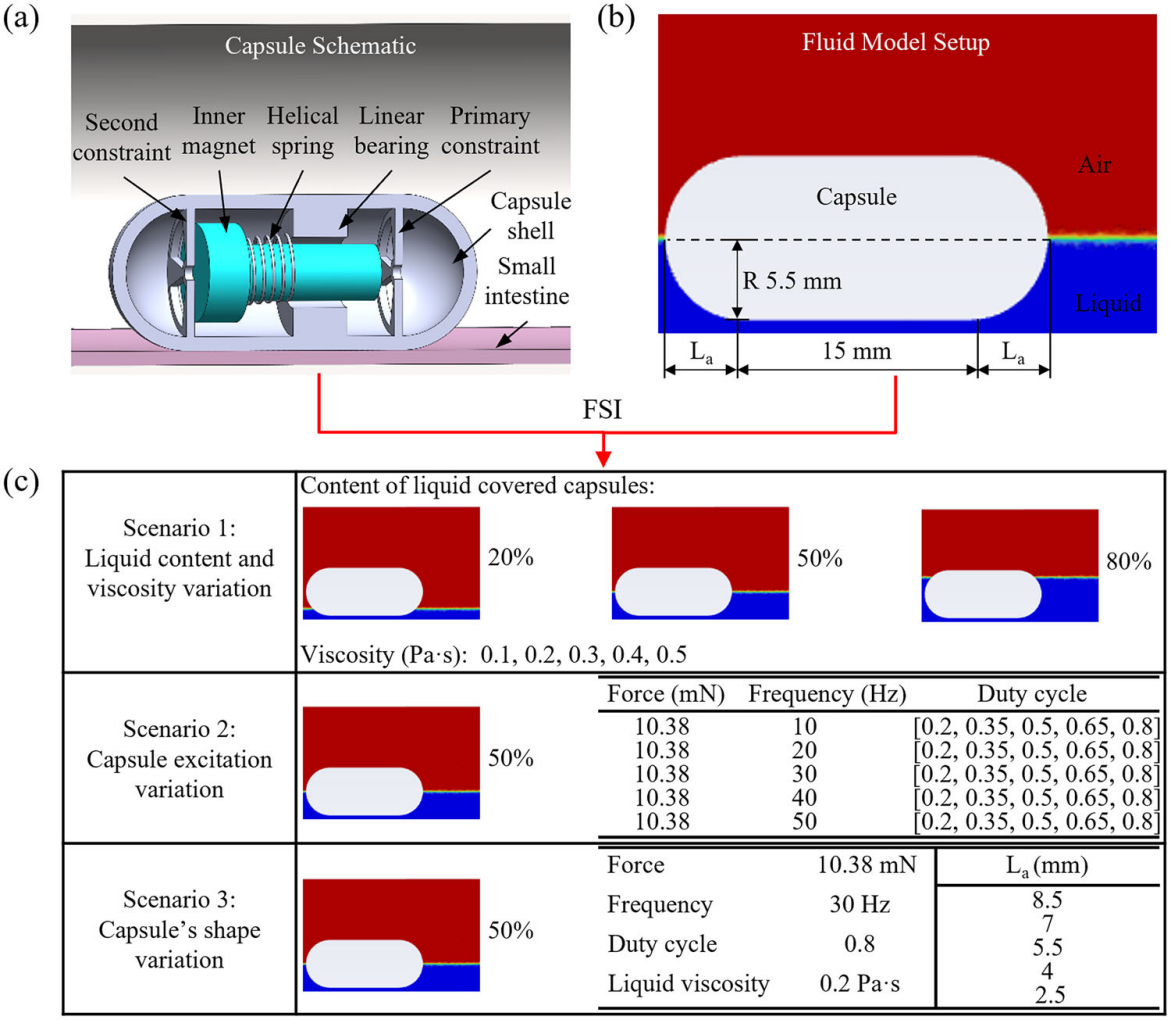
Schematic illustration of the modelling framework, including (a) the capsule-intestine structural model, (b) the fluid domain setup, and (c) the three simulation scenarios considered in this study. The capsule-intestine structural model shows the inner structure of the vibro-impact capsule, including the inner magnet, helical spring, linear bearing, and both primary and secondary constraints within the capsule shell. An artificial small intestine is placed beneath the capsule to mimic the physiological boundary conditions. The fluid flow model presents the setup of the two-phase (gas-liquid) fluid model, where the capsule (radius R = 5.5 mm) is partially submerged in artificial fluid, simulating the intestinal environment *in vivo*. The three simulation scenarios analysed in this study: 1) variation of liquid volume fraction and viscosity, where the liquid covers 20%, 50% and 80% of the capsule height, and the viscosity is set to 0.1, 0.2, 0.3, 0.4, or 0.5 Pa$\cdot$s; 2) variation of capsule excitation parameters, with the liquid covering 50% of the capsule and the actuation force fixed at 10.38 mN, while the excitation frequency is set to 10, 20, 30, 40 and 50 Hz, and the duty cycle is varied among 0.2, 0.35, 0.5, 0.65, and 0.8; 3) variation of capsule end geometry, also with 50% liquid coverage, where the capsule end axial length ($L_\text {a}$) is set to 8.5, 7, 5.5, 4, or 2.5 mm, while the excitation parameters are fixed at a frequency of 30 Hz, a duty cycle of 0.8, and a liquid viscosity of 0.3 Pa$\cdot$s

### Geometrical modelling and analysis strategy

The bidirectional FSI model couples a structural simulation of the capsule-intestine interaction with a fluid simulation of the surrounding intestinal environment, as shown in Figs. 4a and b, respectively. In Fig. 4a, the capsule size is consistent with the experiment and has a shell with a semicircular front and rear shape, which contains an inner propulsion system composed of linear bearings, coil springs, the inner magnet and dual constraint mechanisms (primary and secondary constraints). When the component is subjected to external magnetic excitation, the magnet compresses the spring, and it is not excited; the spring rebounds and the magnet impacts the primary and secondary constraints. The periodic square-wave signal can cause the magnet to produce periodic vibration and reciprocating motion and cause the capsule to move. In Fig. 4b, the fluid domain includes a simplified part of the small intestine, represented as a rigid tube, partially filled with liquid to simulate intestinal contents. Above the liquid phase, air is modelled, and an air exchange port is set up to consider potential two-phase dynamics.

In order to analyse the impact of key factors on capsule propulsion, three simulation scenarios were established. Scenario 1: Change the liquid volume fraction (20%, 50%, 80% capsule coverage) and viscosity (0.1-0.5 Pa$\cdot$s) to evaluate their combined effects on resistance and propulsion force. Scenario 2: Changes in the excitation parameters of the capsule, including the driving force, frequency (10-50 Hz), and duty cycle (0.2-0.8), under constant liquid coverage 50%. Scenario 3: Changing the geometry of the capsule by adjusting the length of the axial radius at both ends ($L_\text {a}$) while keeping all other parameters constant, to investigate the effect of shape on fluid interaction and propulsion. These scenarios were designed to reflect clinically relevant conditions, such as variations in fluid content and excitation parameters, while remaining compatible with experimental setups, enabling a systematic investigation of FSI behaviour.

### Material description

A critical aspect of accurately simulating the capsule-intestine interaction in the FSI model is the realistic representation of intestinal tissue mechanics. The small intestine consists of several anatomically and functionally distinct layers, including the serosa, longitudinal muscle, circular muscle, submucosa, and mucosa [49]. Each layer contributes differently to the overall mechanical response, with the muscle layers dominating the active contractile properties and the submucosa and mucosa imparting additional compliance and damping. The small intestine used in this study is synthesized from human tissue analogs and woven fibers, which can closely mimic the material properties of the real small intestine. Therefore, in the present study, the artificial small intestine is characterised as a viscoelastic material, reflecting both its elastic and viscous properties under physiological loading. The time-dependent response of the intestine wall to stress and strain is characterised by the relaxation modulus. The relaxation modulus describes the reduction of internal stress in tissue over time under constant strain, capturing the essence of viscoelasticity. According to our previous measurements [27], a three-parameter Maxwell model was used in this model to describe the viscoelastic response of the intestinal wall and simulate the capsule-intestine interaction. The Maxwell model with three parameters combines a spring (elastic element) and two dampers (viscous element) to simultaneously capture the instantaneous elastic response and delayed viscous relaxation of the tissue, and its constitutive equation used in the FE model is given as

10 $$\begin{aligned} \sigma (t) = \epsilon (E_\text {i} e^{-\frac{E_\text {i}}{\eta _\text {i}} t } + E_\infty ), \end{aligned}$$

where $\sigma$ represents circumferential stress and $\epsilon =(R_\text {i}-R_\text {c})/R_\text {i}$ represents intestinal strain. $R_\text {c}$ and $R_\text {i}$ are the outer radius of the capsule and the inner radius of the intestine. $E_\text {i}$, $E_\infty$ and $\eta _\text {i}$ represent the Young’s modulus of the spring and the viscosity coefficient of the damper. The ratio of $\eta _\text {i}$ to $E_\text {i}$ is the delay, representing the time range in which the modulus of a series of single spring dampers decreases from $E_\text {i}$ to 0, and the relaxation time $\tau$. The ratio of $E_\text {i}$ to the transient relaxation modulus $E_0 = E_\text {i} + E_\infty$ is the relative modulus, which has been implemented as a standard input of viscoelastic material in software and is represented by the Prony function series.

**Table 1:** Material parameters of FSI model

Material Parameters	Value	Unit	Material Parameters	Value	Unit
$E_\text {i}$	0.196	MPa	$\rho _1$	1.01	$\mathrm {g/cm}^3$
$E_\infty$	0.757	MPa	$\rho _2$	1.04	$\mathrm {g/cm}^3$
$E_\text {c}$	1100	MPa	$\rho _3$	1.06	$\mathrm {g/cm}^3$
$\nu _\text {i}$	0.49	-	$\rho _4$	1.08	$\mathrm {g/cm}^3$
$\nu _\text {c}$	0.42	-	$\rho _5$	1.09	$\mathrm {g/cm}^3$
$\eta _\text {i}$	5.36	MPa$\cdot$s	$\eta _1$	0.1	Pa$\cdot$s
$\eta _\text {g}$	0.018	MPa$\cdot$s	$\eta _2$	0.2	Pa$\cdot$s
$\rho _\text {i}$	1	$\mathrm {g/cm}^3$	$\eta _3$	0.3	Pa$\cdot$s
$\rho _\text {c}$	0.95	$\mathrm {g/cm}^3$	$\eta _4$	0.4	Pa$\cdot$s
$\rho _\text {g}$	0.00125	$\mathrm {g/cm}^3$	$\eta _5$	0.5	Pa$\cdot$s

The capsule’s propulsion mechanism relies on an internal magnet that is periodically actuated by a square wave signal applied to an external electromagnetic coil. During each cycle, the magnet is driven to collide alternately with the primary and secondary internal constraints via a spring mechanism. When the external signal is off (zero), the spring returns the magnet toward the opposite constraint. This reciprocating impact generates vibro-impact forces that result in net propulsion of the entire capsule [29]. A simplified dynamic model of this mechanism, accounting for the capsule’s interaction with the fluid environment, is given by

11 $$\begin{aligned} {\left\{ \begin{array}{ll} m_\text {m} \ddot{x}_\text {m} = F - F_\text {i},\\ m_\text {c} \ddot{x}_\text {c} = F_\text {i} + F_\text {x} + F_\text {f}, \end{array}\right. } \end{aligned}$$

where $m_\text {m}$ is the mass of the internal magnet, $m_\text {c}$ is the mass of the capsule shell, $\ddot{x}_\text {m}$ and $\ddot{x}_\text {c}$ are the accelerations of the magnet and capsule shell, respectively. *F* is the magnetic excitation force given by Eq. (8), $F_\text {i}$ is the interaction (impact) force between the magnet and the capsule shell, $F_\text {f}$ is the Coulomb friction force between the capsule and the intestinal wall, and $F_\text {x}$ represents the hydrodynamic resistance from the surrounding fluid. This model captures the essential momentum transfer mechanism through which internal impact events generate the forward and backward motion of the capsule. A more detailed explanation of this model is provided in [23].

The viscosity of intestinal fluids is highly variable and can increase markedly in pathological states. In particular, excessive secretion and accumulation of mucus, commonly observed in inflammatory bowel disease, infection, or partial obstruction, can lead to local viscosities ranging from 0.1 to 0.5 Pa$\cdot$s or even higher [52]. Based on these findings, the viscosity range of artificial intestinal fluid in this study was set to 0.1-0.5 Pa$\cdot$s to represent challenging conditions characterised by mucus thickening or other pathological changes. This selection aligns with established precedents in [53], enabling a comprehensive evaluation of capsule propulsion under increased luminal resistance, as may be encountered in certain patient populations. To improve computational efficiency and facilitate direct comparison with previous studies, artificial intestinal fluid was modelled as a Newtonian fluid. The Newtonian approximation is widely accepted within the studied viscosity range and the relevant experimental shear rates, and its validity has been confirmed in related research [26, 43, 54, 55]. Artificial intestinal fluid was prepared by mixing the lubricant with water in specific ratios to achieve target viscosities of 0.1, 0.2, 0.3, 0.4, and 0.5 Pa$\cdot$s. The corresponding fluid densities used in the simulation are summarised in Table 1. The gas phase in the model represents the gaseous contents of the intestinal lumen. The composition of intestinal gases has been reported to closely resemble that of ambient air, with nitrogen, oxygen, and trace gases being the dominant constituents. Consequently, standard atmospheric values were used for gas density $\rho _\text {g}$ and viscosity $\eta _\text {g}$ in the simulation.

Furthermore, Table 1 provides detailed properties of the material relevant to the FSI model, including the density $\rho _\text {i}$ and the Poisson ratio $\nu _\text {i}$ of the small intestine, the Young modulus $E_\text {c}$, the Poisson ratio $\nu _\text {c}$ and density $\rho _\text {c}$ of the capsule shell, and the densities ($\rho _1 - \rho _5$) and the viscosity ($\eta _1 - \eta _5$) corresponding to the five different artificial intestinal fluids evaluated in this study.

### Configurations of fluid-structure interaction model

The bidirectional FSI model couples both the structure simulation and the fluid flow simulation. In the transient structural module, it was established by importing the geometries of the capsule shell, the magnet, and the wall of the small intestine. The total mass of the capsule was set at 4.88 g, corresponding to the experimental prototype. The permanent magnet was mounted along the horizontal axis of the capsule and was defined with translational freedom to allow a realistic dynamic response. The linear spring connecting the magnet and the capsule shell was simplified using a one-dimensional axial tension-compression element (COMBIN14), with a longitudinal stiffness of 0.0625 N/mm and damping coefficient of 0.0156 N$\cdot$s/m. Contact interactions between the capsule and the intestinal wall were defined with a friction coefficient of 0.02 [56], and the effect of gravity was also included in the model. The inner magnet excitation was implemented as a time-dependent force, controlled by the frequency and duty cycle of the external square wave signal, which corresponded to the experimental parameters; the maximum excitation force applied was 10.38 mN. The outer surface of the capsule was designated as the FSI interface for data exchange with the fluid solver.

In the fluid domain, only the external contour of the capsule and the surrounding fluid region were retained for simulation. A Volume of Fluid method was used to capture the dynamics of the two-phase gas-liquid flow, with the surface tension at the gas-liquid interface assumed to be that of water (72.75 mN/m). To resolve complex near-wall flow characteristics, including the potential laminar-turbulent transition and the separation of the boundary layer induced by the vibrational motion of the capsule and the effects of the gas-liquid interface, the Shear Stress Transport (SST) $k-\omega$ turbulence model [57] was selected to simulate the intestinal environment. In the operating conditions of this study, the Reynolds number based on the diameter of the capsule and the average velocity is in the range of 100-800, belonging to the laminar to the transitional flow range. The SST $k-\omega$ model exhibits robustness in this interval. We assume that the fluid is incompressible, which is reasonable for both liquids (water-based solutions) and low-speed airflow. To build the FSI model, the following material and modelling assumptions were applied:

- The small intestine is isotropic, uniform, and incompressible;
- The inner magnet can only move along the frictionless bearing in the axial direction of the capsule;
- Intestinal fluid is relatively high viscosity and Newtonian fluid;
- The intestinal gas was assumed to have the same composition as air and to exhibit the surface tension of water at the gas-liquid interface.

The fluid domain adopts an unstructured tetrahedral / polyhedral mesh, the first layer having a height of approximately 0.05 mm, a total of 5 layers, a growth rate of 1.2, and a global maximum mesh size of 2 mm. The boundary layer is densified near the capsule wall, and the mesh is refined to 0.5 mm. The outer contour of the capsule in the fluid module was established as a moving and deforming boundary using a dynamic mesh system coupling approach, which facilitates real-time transfer of fluid forces to the structural module. Both gravity and buoyancy effects were included in the fluid analysis. The entire bidirectional FSI was realised through the system coupling module in the FE software, enabling synchronous solution and data exchange between the structural and fluid solvers.

## Experimental and simulation results

This section presents a comprehensive comparison and analysis of the experimental data and the FSI results for the three defined scenarios. For each scenario, key performance indicators such as capsule displacement, velocity, and interactions with the fluid and the intestinal wall are analysed. The aim is to elucidate the dynamic behaviour of the vibro-impact capsule under different physiological and operational conditions and to validate the predictive capacity of the FSI model against experimental observations.

**Fig. 5:**
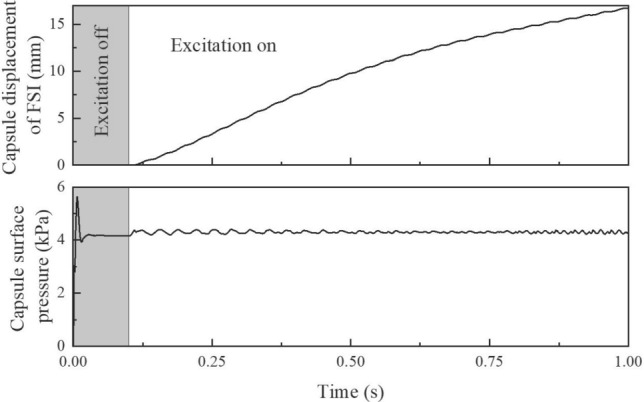
Time history of the capsule displacement and surface pressure in the FSI model. The shaded region ($t<$ 0.1) corresponds to the initial settling phase, during which the capsule is not excited and only gravity and buoyancy are considered. After $t=$ 0.1 s, the vibro-impact capsule is magnetically actuated, leading to rapid displacement growth and a stable surface pressure response

In all FSI models, a consistent initial condition is implemented to closely match the experimental protocol. The time evolution of the displacement and velocity of the capsule is illustrated in Fig. 5. During the first 0.1 seconds of each simulation, no excitation was applied to the capsule. This allowed the capsule to settle under the combined effects of gravity and buoyancy, ensuring that it reached a stable equilibrium position before the onset of magnetic actuation. This approach ensures the comparability of the initial states between simulation and experiment, thereby improving the reliability of the subsequent dynamic analysis.

### Scenario 1: Variation of liquid volume fraction and viscosity

**Fig. 6:**
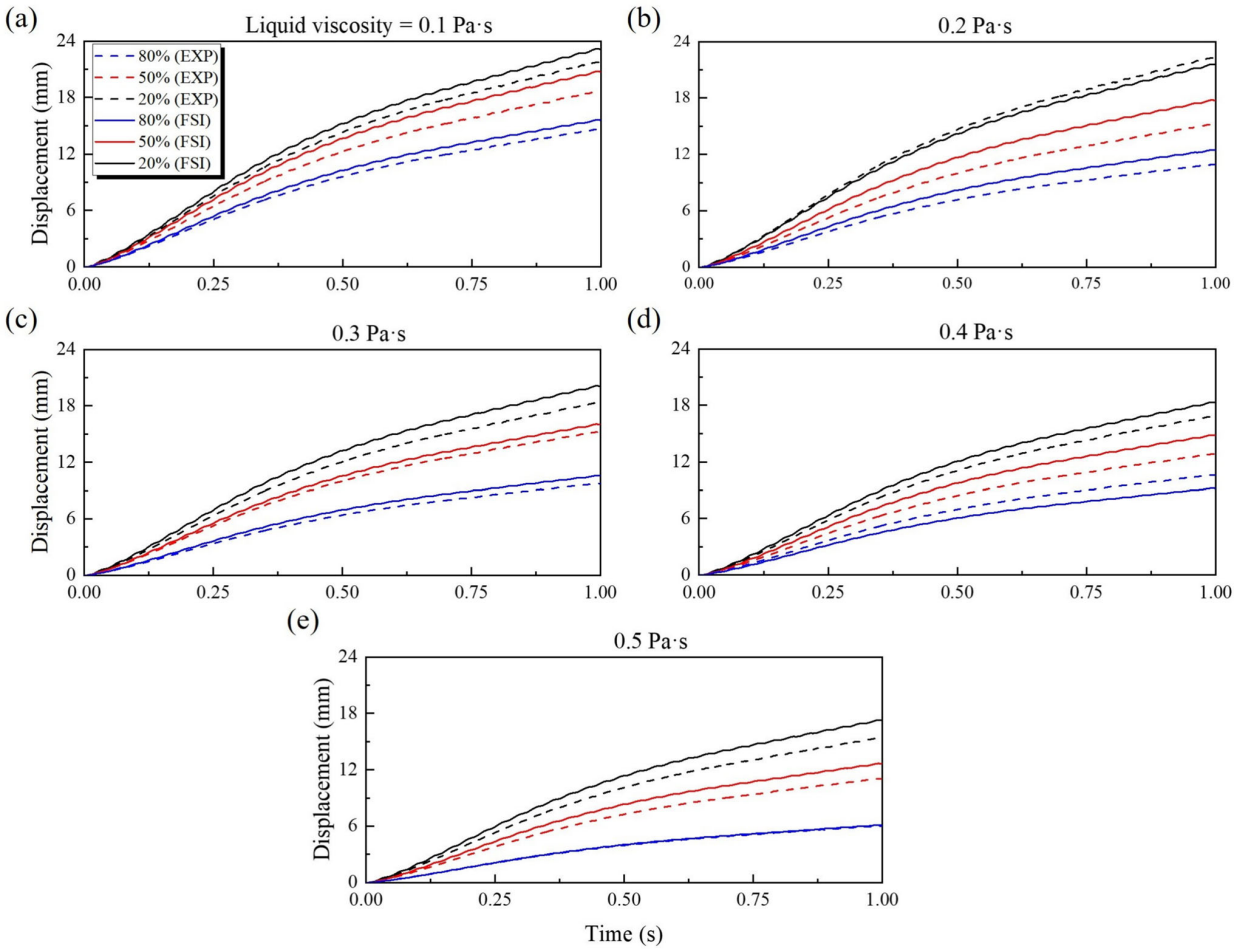
Effects of liquid viscosity and fluid volume fraction on capsule propulsion: Comparison between FSI simulation and experimental results (EXP). (a-e) Displacement of the capsule as a function of time for five liquid viscosities: (a) 0.1 Pa$\cdot$s, (b) 0.2 Pa$\cdot$s, (c) 0.3 Pa$\cdot$s, (d) 0.4 Pa$\cdot$s, and (e) 0.5 Pa$\cdot$s. In each graph, the results are shown for three levels of liquid coverage: 20% (black), 50% (red), and 80% (blue) of the height of the capsule. Solid lines represent FSI simulation results, while dashed lines represent experimental measurements

In Scenario 1, the effects of both the liquid volume fraction and the viscosity of the surrounding fluid on the propulsion of the capsule were investigated. Fig. 6 presents a comprehensive comparison between experimental measurements and FSI simulation results for capsule displacement over time under three liquid coverage conditions (20%, 50% and 80%) and five viscosity values (0.1 to 0.5 Pa$\cdot$s). In addition, the evolution of the maximum pressure exerted on the surface of the capsule for each corresponding case is performed, allowing further insight into the hydrodynamic load experienced by the capsule under varying fluid conditions.

**Fig. 7:**
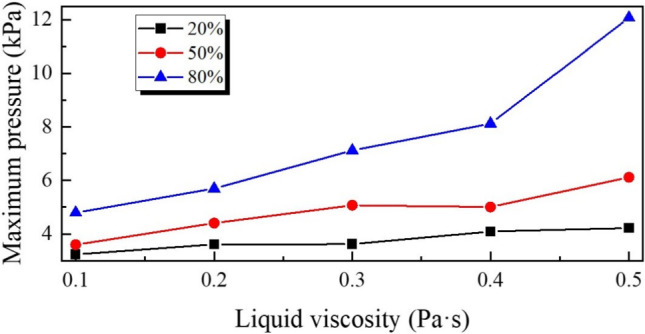
Maximum pressure on the surface of the capsule as a function of the liquid viscosity for the three volume fractions. Symbols indicate the results for the liquid coverage 20% (squares), 50% (circles), and 80% (triangles)

As illustrated in Fig. 6a-e, the FSI simulation results show good general agreement with the experimental measurements, showing only minor discrepancies between all the conditions tested. This close correspondence supports the accuracy and predictive capability of the developed bidirectional FSI model. The results indicate that increasing the liquid volume fraction exerts a substantially greater inhibitory effect on both capsule velocity and displacement compared to increasing viscosity within the examined range. Specifically, as the liquid volume fraction increases from 20% to 80%, there is a marked reduction in the displacement of the capsule during the same actuation period, and the displacement curves for the liquid volume fraction of 20% and 50% are consistently higher than those for 80%. As shown in Fig. 6e, even at the highest tested viscosity (0.5 Pa$\cdot$s), the displacement difference between 20% and 80% remains considerable. However, by comparison among Fig. 6a-e, the differences between the displacement curves at varying viscosities but with constant liquid coverage are comparatively minor. These findings suggest that, under the present experimental conditions, the overall volume of liquid imposes a more significant resistance to capsule movement than viscosity alone. In addition, it is worth noting that the experimental displacement results are generally slightly lower than the FSI predictions. This discrepancy can be attributed to the limitations in manual operation of the external coil during experiments; specifically, it is challenging to perfectly synchronise the coil movement with the actual speed of the capsule, resulting in occasional under-driving or nonideal force transmission. Such factors lead to slightly reduced propulsion performance in experiments compared to idealised simulation. In Fig. 7, it illustrates the maximum pressure acting on the capsule surface during propulsion. As the liquid volume fraction increases, the maximum surface pressure on the capsule increases markedly, reflecting a higher hydrodynamic load. Although increasing the viscosity also increases the maximum surface pressure, its effect is less significant than that of increasing the liquid volume fraction. The steepest increases in maximum pressure correspond to the highest fluid content conditions, further highlighting the dominant effect of liquid volume on both propulsion resistance and surface pressure.

**Fig. 8:**
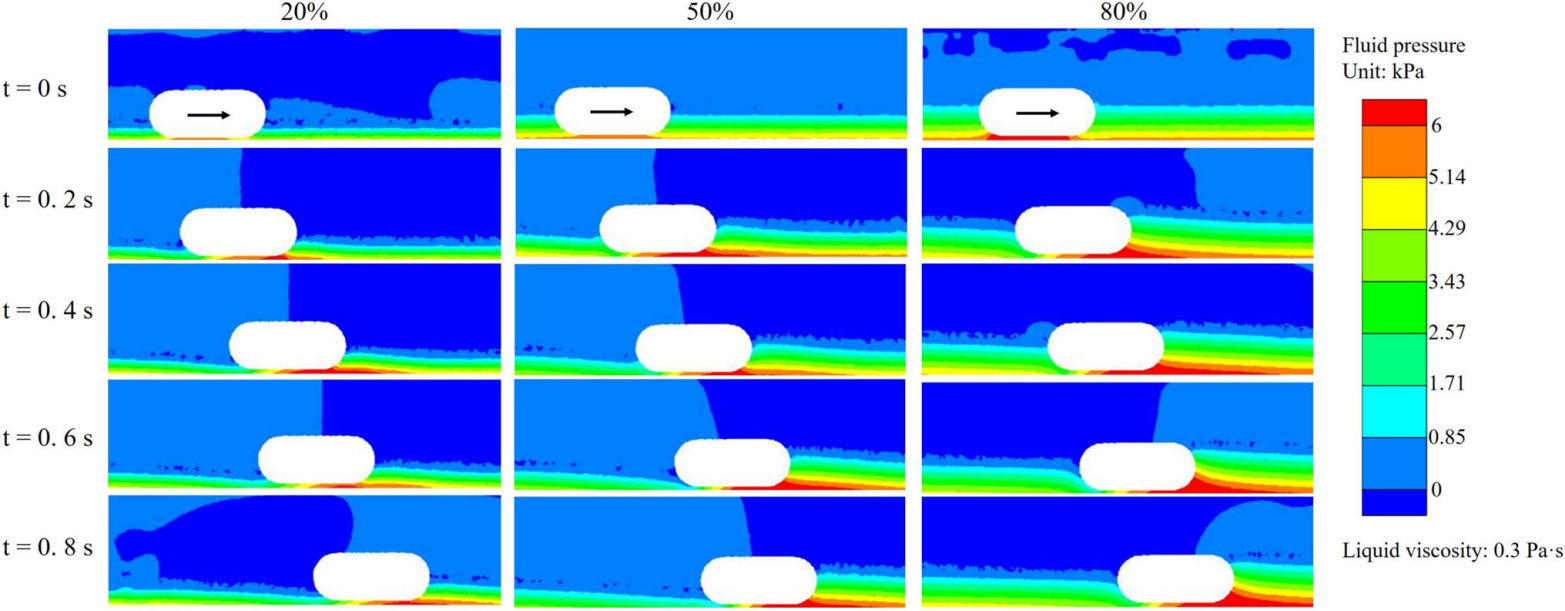
The fluid pressure field around the capsule for different liquid volume fractions at a viscosity of 0.3 Pa$\cdot$s. Each row corresponds to a different fraction of fluid coverage (20%, 50% and 80% of the capsule), while each column represents a specific time point during propulsion: t = 0 s, 0.2 s, 0.4 s, 0.6 s and 0.8 s. The arrow indicates the direction of movement of the capsule. The colour scale indicates the fluid pressure in units of kPa

To further elucidate the hydrodynamic mechanisms underlying the observed reduction in capsule velocity and displacement, Fig. 8 presents fluid pressure contour maps around the capsule at different time points and for varying liquid volume fractions and viscosities. A distinct feature emerging from these contour maps is the pronounced accumulation of liquid at the front (head) of the capsule during propulsion. This accumulation results in a localised region of elevated pressure over 6 kPa acting on the surface of the anterior capsule. As shown, the extent and intensity of this high-pressure region increase substantially with increasing liquid volume fraction. When the volume fraction is low (e.g. 20%), the liquid layer above the capsule remains relatively thin, and the corresponding pressure build-up is modest. In contrast, as the liquid fraction increases to 50% and especially 80%, the volume of fluid that accumulates in front of the capsule increases markedly, generating a broader and more intense high-pressure zone. Furthermore, although not shown graphically, a comparison of different viscosity values reveals that while a higher viscosity slightly increases the overall pressure levels around the capsule, the dominant factor influencing the formation and magnitude of the high-pressure region remains the liquid volume fraction. At any given viscosity, the trend of increased fluid accumulation and pressure build-up with higher liquid volume fraction persists, in agreement with the quantitative displacement and pressure data discussed above.

**Fig. 9:**
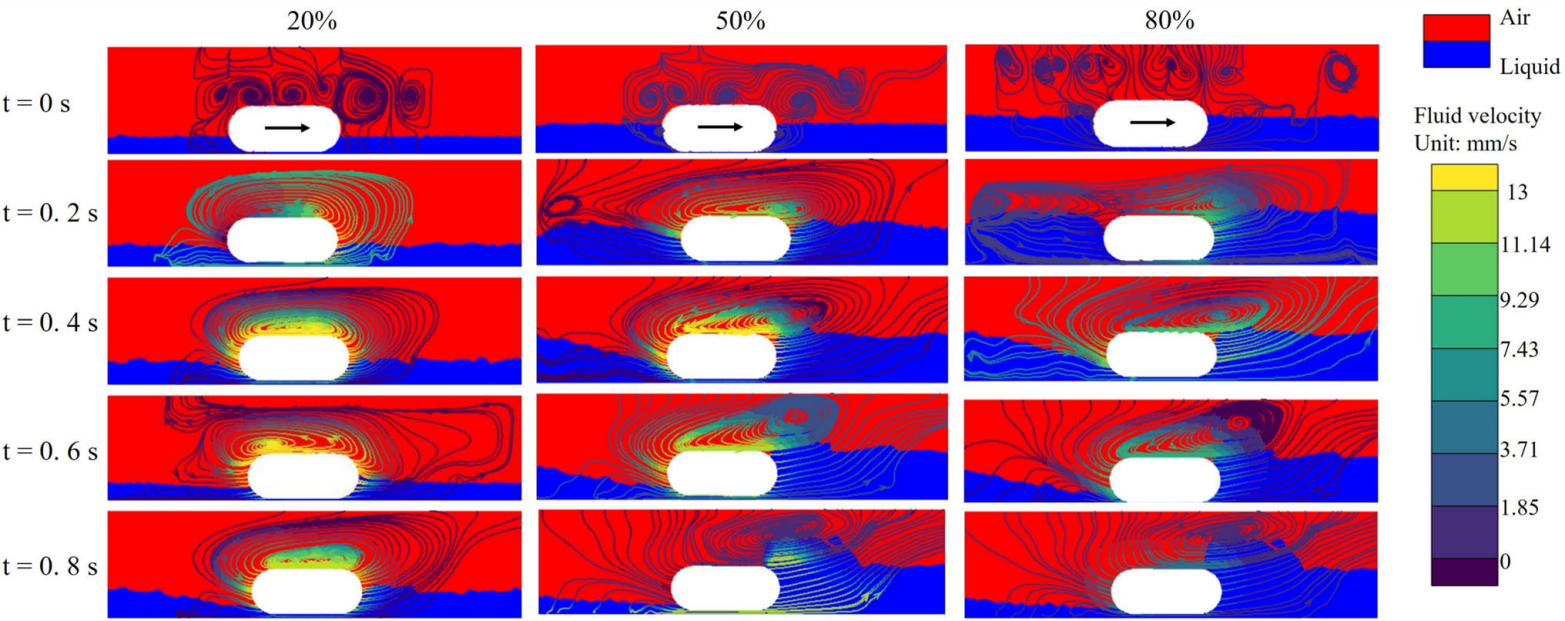
Evolution of flow velocity fields and streamlines for different fractions of liquid volume around the capsule at a fixed viscosity of 0.3 Pa$\cdot$s. Each column corresponds to a specific fluid coverage fraction (20%, 50%, and 80% of the capsule height), while each row shows a distinct time point during propulsion (t = 0 s, 0.2 s, 0.4 s, 0.6 s and 0.8 s). The background colour indicates gas-liquid distribution in the intestine with red representing air and blue representing liquid. The arrow on the capsule indicates the direction of movement of the capsule. The colour lines represent streamlines and different colours indicate their flow velocity

To further validate and visualise the influence of liquid volume fraction on capsule propulsion, Fig. 9 presents the flow field streamlines and phase volume fraction distributions at representative time points for various fluid coverage conditions. In these images, the streamlines illustrate the direction and magnitude of the local fluid velocity, while the background colour map depicts the spatial distribution of the liquid and air phases. From the results, it is evident that as the capsule moves forward, a prominent recirculation zone develops around it, with both the intensity and spatial extent of these vortices increasing under higher liquid volume. At lower fractions of liquid (20%), the vortices are relatively weak and confined, allowing efficient fluid displacement around the capsule and minimal resistance to the forward motion. In contrast, at higher liquid fractions (50% and 80%), the flow streamlines near the capsule front become denser and more distorted, leading to the formation of stronger vortical patterns and localized fluid entrapment. This is accompanied by a thicker accumulation of liquid in front of the capsule, as indicated by the distribution of the phase volume fraction. These flow features are consistent with the pressure field results, highlighting that the formation of large-scale recirculation zones and liquid accumulation acts as a hydrodynamic barrier, significantly impeding capsule propulsion. The increased complexity and strength of these vortical structures at a higher liquid coverage highlight the dominant role of fluid volume in resisting capsule motion.

In summary, both quantitative data and flow field visualisations consistently demonstrate that increasing the liquid volume fraction around the capsule imposes a much greater inhibitory effect on propulsion than increasing viscosity alone. The bidirectional FSI model agrees well with the experimental observations, with only minor discrepancies attributable mainly to manual control limitations in the experimental setup. The pressure field and streamline analyses reveal that the high liquid volume fraction promotes pronounced fluid accumulation and vortex formation at the front of the capsule, resulting in elevated hydrodynamic resistance and decreased displacement. These findings highlight the critical importance of fluid volume fraction in governing the locomotion performance of the capsule within liquid-filled intestinal environments.

### Scenario 2: Variation of capsule excitation

**Fig. 10:**
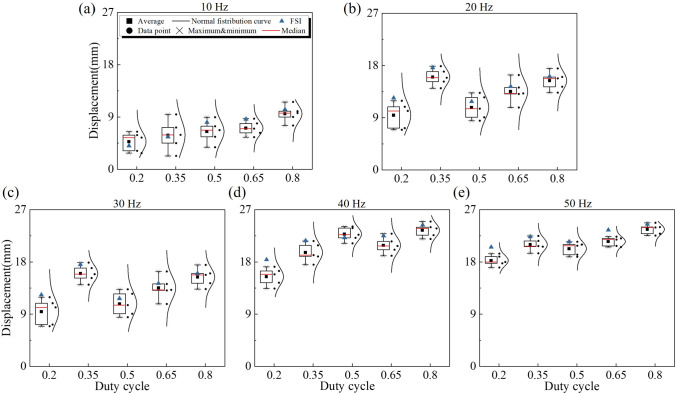
Comparison of experimental and FSI simulation results of the capsule displacement at different actuation duty cycles and frequencies. (a) 10 Hz and (b) 20 Hz. All cases correspond to a fixed liquid viscosity of 0.3 Pa$\cdot$s and 50% liquid volume fraction that covers the capsule, with displacement measured after 1 second of actuation. For each condition, box plots show the experimental data, with black squares denoting the mean, red lines the median, $\times$ the maximum and minimum, black dots the individual data points, and the surrounding curve the normal distribution fit. The blue triangles represent the corresponding FSI simulation results

Scenario 2 presents a comparative analysis of the experimental data and FSI results of the capsule displacement at different excitation frequencies and duty cycles, with all tests conducted at a fixed viscosity of 0.3 Pa$\cdot$s and a liquid coverage of 50% of the capsule volume. It is worth to notice that all results are obtained from capsule displacement achieved after 1 second of magnetic actuation. As shown in In Fig. 10, the box plots and accompanying normal distribution curves illustrate the distribution of experimental measurements, including average, median, and extreme values, while blue triangles denote the FSI results under the corresponding excitation parameters. All FSI results show a good agreement with the average values of the experimental data, and minor deviations may be due to experimental bias and actuation timing variations. These results not only validate the predictive accuracy of the FSI model but also highlight the robustness of the FSI model.

Across all frequencies, the displacement of the capsule exhibits a clear upward trend with an increasing duty cycle, especially as shown in Fig. 10a, d and e. In addition, as the frequency increases from Fig. 10a to e, the moving distance of the capsule gradually increases at the same duty cycle. The fastest capsule movement speed occurs at 40Hz, 80% duty cycle and 50 Hz, 80% duty cycle. This shows that increasing the excitation frequency improves the propulsion efficiency of the capsule in a fluid environment. Moreover, the variance among the experimental results decreases as both the duty cycle and the frequency increase, implying that the capsule’s motion becomes more consistent and controllable with stronger and more sustained excitation. For example, at 50 Hz and a duty cycle of 0.8, the normal distribution curve becomes narrower and more centred, reflecting that the capsule motion is less susceptible to random disturbances under high-frequency and high-duty cycle actuation.

In summary, the bidirectional FSI model matches well with the experimental data and provides reliable insight into the parameter space of magnetic actuation. The findings of this scenario indicate that optimising the frequency and duty cycle is critical to achieving efficient and predictable capsule propulsion, which is essential for practical *in vivo* applications.

### Scenario 3: Variation of capsule shape

In this scenario, the effect of the capsule shape, specifically the axial semi-radius length ($L_\text {a}$) shown in Figs. 4b, on the propulsion performance was investigated. All tests were carried out under fixed actuation and fluid conditions, including an excitation frequency of 30 Hz, a duty cycle of 80%, an excitation amplitude of 10.38 mN, a liquid viscosity of 0.2 Pa$\cdot$s, and 50% liquid coverage of the capsule volume. The only variable parameter in this set of experiments and simulations was the $L_\text {a}$, which was varied among five values: 2.5, 4, 5.5, 7, and 8.5 mm.

**Fig. 11:**
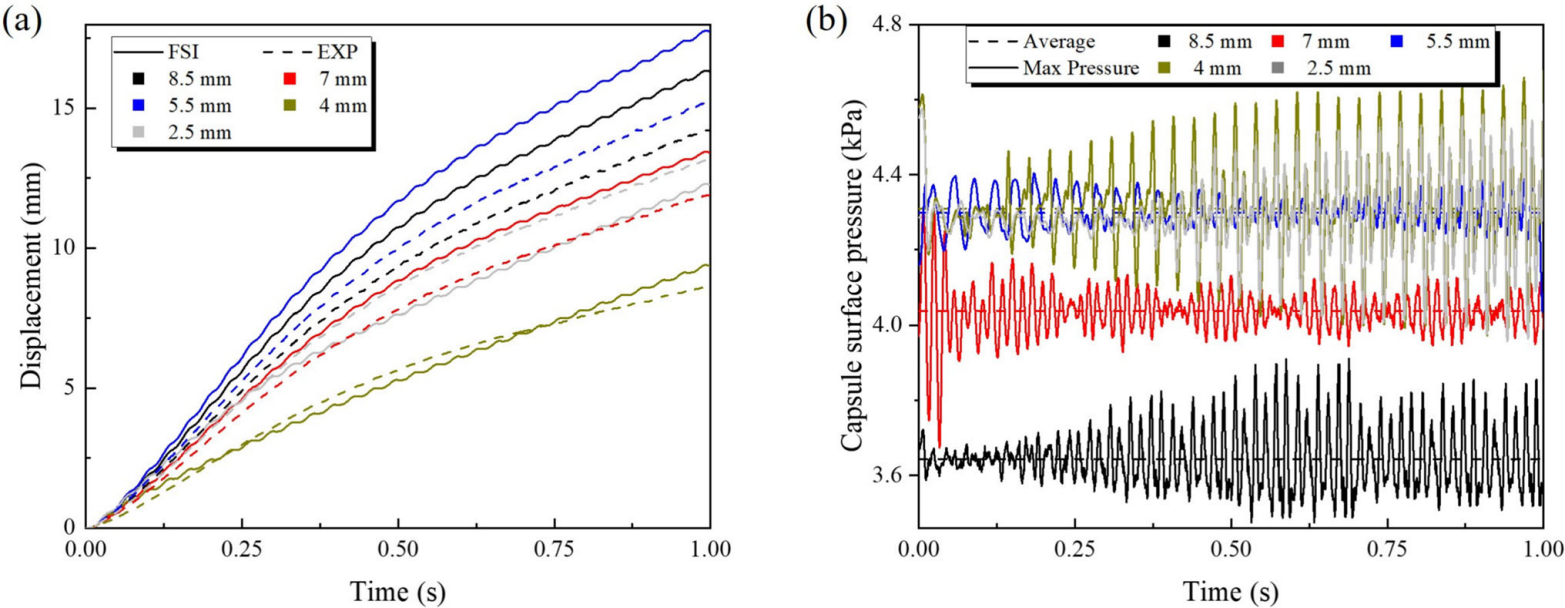
Time histories of (a) capsule displacement and (b) surface pressure for five different capsule end radii ($L_\text {a}$ = 8.5, 7, 5.5, 4 and 2.5 mm), under the same actuation parameters: 30 Hz frequency, 0.8 duty cycle, 10.38 mN actuation force, 0.2 Pa$\cdot$s liquid viscosity, and 50% liquid volume fraction. Displacement curves from FSI simulations and experiments show that the capsule with standard semicircular end with 5.5 mm radius achieves the largest displacement, while others lead to reduced propulsion. The pressure curves show a constant average surface pressure and a fluctuating maximum surface pressure on the capsule over time

As a result, Fig. 11a presents the time histories of the capsule displacement for each geometry, based on experimental investigations and the FSI simulations. Notably, the capsule with an end radius of 5.5 mm achieves the highest displacement, while the one with 4 mm shows the smallest. The intermediate values follow the order: 8.5, 7, and 2.5mm. Fig. 11b shows the pressure on the surface of the each capsule during propulsion, based on the FSI simulation results. The results reveal that although capsules with longer ends experience the relative low pressure, the greatest displacement does not correspond to the lowest surface pressure. Instead, a standard semicircular end with 5.5 mm radius appears to achieve the most efficient propulsion. In addition, the capsule with shorter ends lead to higher pressure, which in turn may impede its propulsion.

**Fig. 12:**
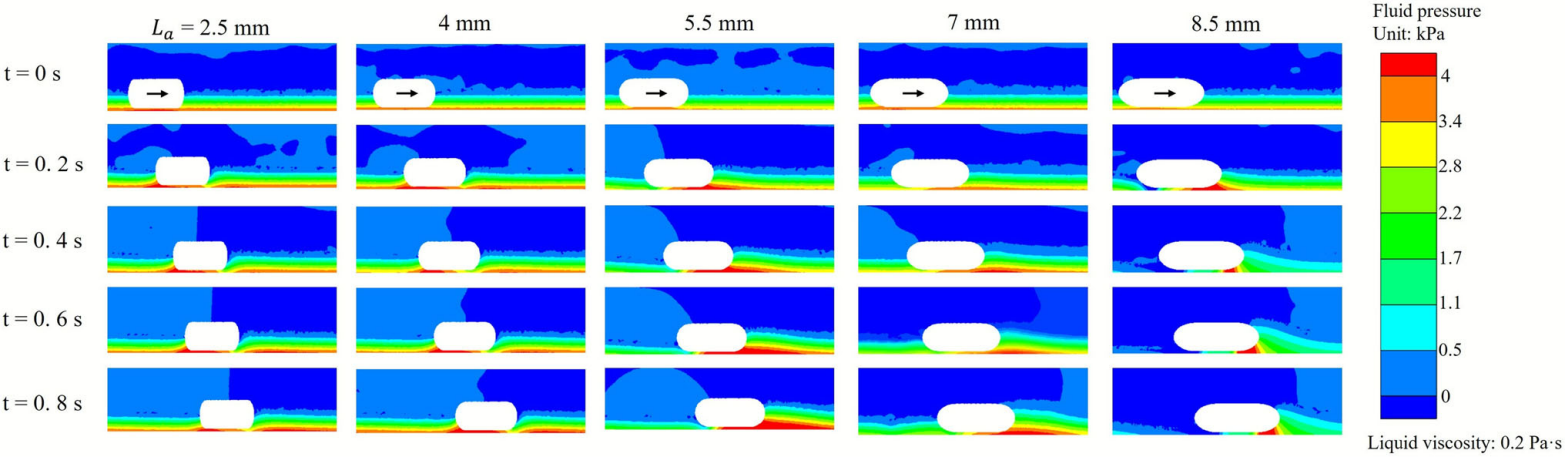
Evolution of the time of fluid pressure fields around capsules with different capsule end radii at a 0.2 Pa$\cdot$s liquid viscosity and at 50% liquid volume fraction. Each row presents results for a different axial radius of the end of the capsule, from left to right: 2.5, 4, 5.5, 7 and 8.5 mm. Each column shows a different time point during the simulation, specifically at 0, 0.2, 0.4, 0.6, and 0.8 s. The arrow indicates the direction of movement of the capsule. The colour contours represent the distribution of fluid pressure in kPa around the capsule as it moves through the fluid

To further elucidate the influence of the geometry of the end of the capsule on hydrodynamic loading, Fig. 12 presents time-resolved contour maps of the liquid pressure around the capsule for various end radii ($L_\text {a}$) during propulsion. These pressure fields provide direct visual evidence of how changes in end geometry affect the spatial distribution and concentration of fluid forces acting on the capsule. A distinct trend is apparent in all cases: for capsules with very short ends ($L_\text {a}$ = 2.5 mm), the liquid pressure is predominantly concentrated in the rear of the capsule during motion. This concentration at the tail may be attributed to increased flow separation and recirculation in the wake of the blunt end, resulting in a localised build-up of hydrodynamic pressure that resists forward movement. In contrast, as the end of the capsule becomes longer and more streamlined, the region of high pressure gradually shifts toward the front of the capsule. For the most elongated and tapered capsules, the maximum pressure appears at the leading edge. This redistribution of pressure corresponds to a more streamlined interaction with the surrounding fluid, reducing wake formation and allowing the capsule to cut more efficiently through the liquid. However, as previous results have shown, optimal locomotion performance does not occur in the most extremely elongated shape, but rather at an intermediate geometry (5.5 mm), where pressure is favourably distributed and overall resistance is minimised. Furthermore, the intensity and spread of the high-pressure zone differ between the geometries. Although a concentrated pressure peak is observed at the head for sharply pointed capsules, blunter capsules exhibit broader regions of moderate pressure, particularly around the tail and lateral surfaces. This broader distribution also contributes to increased drag and reduced displacement.

**Fig. 13:**
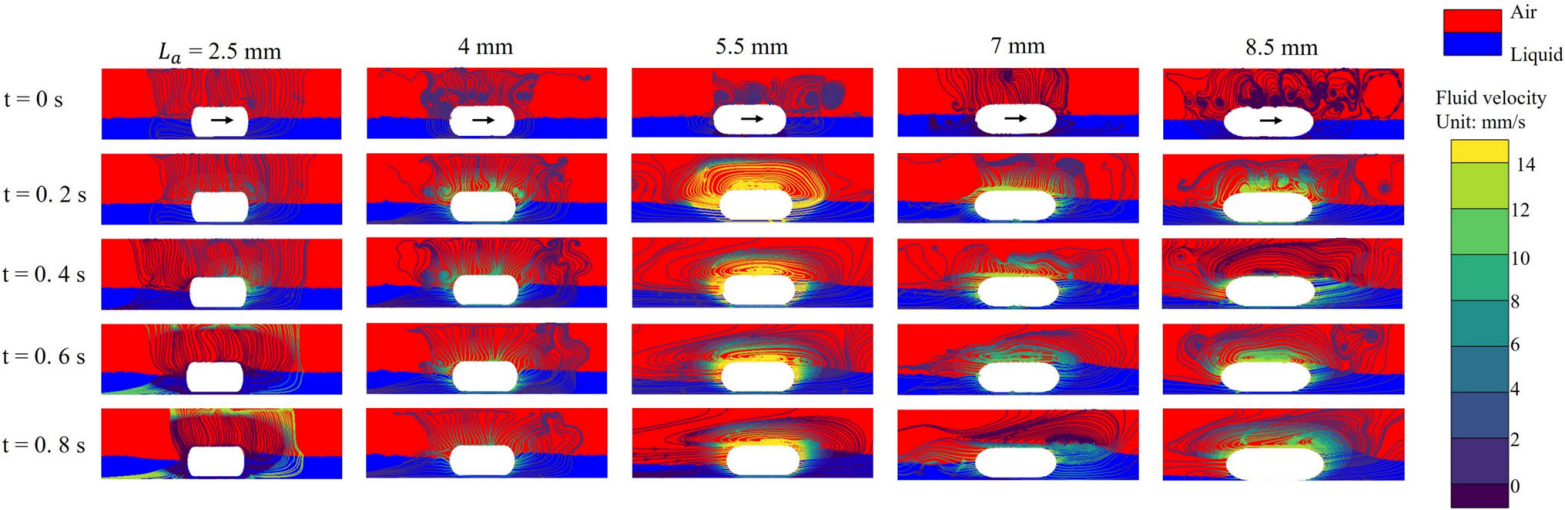
Evolution of transient velocity fields and streamlines for capsules with five different capsule end radii (2.5, 4, 5.5, 7 and 8.5 mm, arranged from left to right), under the condition of 0.2 Pa$\cdot$s liquid viscosity and 50% liquid volume fraction. Each column presents a snapshot at a specific time: 0, 0.2, 0.4, 0.6, and 0.8 s. The blue regions represent liquid, while the red regions indicate air phase. The velocity magnitude of the fluid is indicated by the colour scale on the right, and the superimposed streamlines depict the direction and strength of the flow field. The arrow on the capsule indicates the direction of movement of the capsule

In addition, to interpret the relationship between the end geometry of the capsule and the propulsion efficiency, Fig. 13 presents the flow velocity streamlines and phase distributions around the capsule for different end radii. These images provide direct insight into how flow structure and vortex dynamics are altered by changes in capsule geometry. For capsules with the shortest end radii ($L_\text {a}$ = 2.5 mm), the flow field is dominated by pronounced wake regions and extensive recirculation zones forming behind the capsule. The streamlines reveal large vortical structures and flow separations at the tail, which correspond well to the regions of high pressure identified in the previous pressure contour maps. This substantial recirculation at the rear of the capsule acts as a drag source, impeding forward motion, and resulting in the lowest displacements observed for these geometries. As the ends of the capsule become more elongated and streamlined, a significant change in the flow pattern is observed. For moderate and long end radii (5.5, 7 and 8.5 mm), the recirculation zones decrease in size and move forward, with the flow streamlines wrapping more smoothly around the capsule. In particular, for the 5.5 mm, which demonstrated optimal displacement in previous analyses, the flow remains attached to the capsule surface for a longer distance, resulting in reduced wake formation and overall lower flow separation. The vortices are less intense and more localised near the leading edge, which not only decreases pressure drag, but also enhances efficient propulsion. In addition, for the most streamlined capsules (8.5 mm and 7 mm), while the wake is minimised and the rear flow is clean, the forward shift of the high-velocity and vortex regions may not always correspond to further improvement in displacement. This finding aligns with previous observations that, beyond a certain level of elongation, further streamline does not significantly enhance propulsion and may even begin to reduce performance, likely due to increased wetted surface area or altered force transmission to the surrounding fluid.

In summary, both pressure and flow field analyses demonstrate that the geometry of the end of the capsule has a significant impact on the hydrodynamic resistance and propulsion efficiency. Blunt or short ends cause increased wake, increased rear pressure, and strong recirculation zones, resulting in increased drag and lower displacement. In contrast, the capsule featuring a standard semicircular end of 5.5 mm radius achieve optimal propulsion by minimising flow separation and distributing pressure more favourably. However, excessive elongation of the ends does not provide additional benefit and may even reduce performance. These findings highlight that the capsule’s end geometry should be neither too blunt nor too sharp, and that optimal propulsion performance in viscous fluids is achieved with a standard semicircular end.

## Conclusion and future work

In this study, a comprehensive bidirectional FSI model was developed for a vibro-impact capsule navigating a fluid-filled intestinal environment. The model incorporates detailed representations of the capsule’s internal structure, active magnetic actuation system, viscoelastic intestinal wall, and multiphase (gas–liquid) flow field, all implemented within a finite element analysis framework (ANSYS 2023 R1). The model’s predictions were validated against systematic experimental results, demonstrating strong agreement across all scenarios. Minor deviations were attributed primarily to manual actuation variability and measurement uncertainties. The combined use of quantitative displacement analysis, pressure distribution mapping, and flow field visualisation provided a thorough understanding of the FSI governing capsule locomotion in complex intestinal environments.

Three representative scenarios were explored through both simulation and experimentation. First, variations in fluid volume fraction and viscosity revealed that increased liquid content had a more pronounced inhibitory effect on propulsion than viscosity alone. The FSI model accurately captured these trends, reflecting both displacement and pressure responses across varying fluid conditions. Flow field analysis further showed that larger fluid volumes promoted fluid accumulation and vortex formation in front of the capsule, increasing hydrodynamic resistance and decreasing propulsion efficiency.

Second, excitation parameters were varied, revealing that higher actuation frequency and duty cycle led to improved propulsion performance and greater motion stability. High-frequency, high-duty-cycle actuation reduced variability in the experimental data, indicating enhanced robustness against external disturbances. The FSI model reliably reproduced these behaviours, confirming its predictive capability under different driving conditions.

Third, the capsule’s geometric configuration, particularly the shape of its ends, was found to significantly affect propulsion. Capsules with moderately streamlined ends exhibited optimal flow patterns and pressure distribution, achieving greater displacement. In contrast, blunt or overly elongated designs increased resistance and diminished propulsion, highlighting the importance of balanced geometric design.

Despite the promising results, several limitations point to avenues for future research. While the Newtonian fluid assumption proved sufficient within the tested range, actual intestinal fluids, particularly in pathological states, may exhibit shear-dependent, non-Newtonian properties. Future work will incorporate non-Newtonian mucus models to enhance physiological relevance. Additionally, the current model treats the intestinal wall as a homogeneous viscoelastic structure. Incorporating layered anisotropic features and active peristaltic motion could yield more accurate FSI predictions and better reflect *in vivo* conditions.

Building on this validated FSI framework, future efforts will focus on optimising capsule design and real-time control strategies. Given the current assumption of a straight intestinal tube in the FSI model, future work will extend the framework to more anatomically realistic geometries that incorporate bends, folds, and variable cross-sections. This will enable the investigation of capsule passability and navigation strategies in complex GI environments. In parallel, integration with external magnetic positioning systems, image-based feedback, and multi-coil electromagnetic actuation arrays will allow for dynamic adjustment of the magnetic field direction and intensity via closed-loop control algorithms. These enhancements aim to guide the capsule along predefined trajectories or maintain its position within specific intestinal segments for detailed diagnostic tasks. Furthermore, simplified parametric or surrogate models, such as regression-based or AI-driven approaches, will be developed using the extensive simulation data generated by the current FSI platform. These models will facilitate rapid design optimisation and support real-time control strategy development. In future extensions of this work, additional performance indicators, such as time-resolved force profiles and propulsion efficiency metrics, will be considered to enhance the quantitative interpretation of simulation and experimental results. These metrics will support the development of surrogate models and facilitate comparative evaluation across different design and control scenarios.

## Data Availability

The computational and experimental data sets generated and analysed during the current study are available from the corresponding author on reasonable request.
